# Potential Distribution Pattern and Ecological Suitability Analysis of *Hippophae Tibetana* Schltdl in China Based on the MaxEnt Model

**DOI:** 10.1002/ece3.72926

**Published:** 2026-01-08

**Authors:** Tao Ma, Dan Yong, Danping Xu, Zhipeng He, Zhihang Zhuo

**Affiliations:** ^1^ College of Life and Environment Sciences HuangShan University Huangshan China; ^2^ College of Life Science China West Normal University Nanchong China

**Keywords:** China, climate change, *Hippophae tibetana*, MaxEnt model, suitable habitat distribution

## Abstract

*Hippophae tibetana* Schltdl, a valuable plant with significant edible, medicinal, and ecological restoration functions, has long attracted considerable attention. This study, based on the MaxEnt model, combines current and future climate scenarios (2050s and 2070s) to predict the distribution of suitable habitats for *H. tibetana* . The results demonstrated that the spatial distribution patterns of *H. tibetana* are primarily governed by the combined effects of key environmental factors, including elevation gradient, annual precipitation variation, and mean annual temperature fluctuation. Modeling results demonstrate that *H. tibetana* currently occupies 157.62 × 10^4^ km^2^ of suitable habitats, showing high concentration in three key zones: (1) Qinghai's eastern‐southwestern belt, (2) Gansu's southeastern/Sichuan's western‐southeastern corridor, and (3) Tibet's eastern‐southwestern quadrant. Under future climate scenarios, with increasing temperatures and changes in precipitation patterns, the suitable habitats for Hippophae are generally expected to expand northeastward, particularly in high‐altitude and northwestern regions where the environment becomes more favorable. However, in some extreme climate scenarios, significant changes in temperature and precipitation could have negative effects on the growth and expansion of *Hippophae*. The study suggests that climate change may drive *H. tibetana* to expand into more suitable areas, but it may also lead to the reduction or migration of suitable habitats in some regions. Therefore, future ecological conservation and planting plans for Hippophae should fully consider the impact of climate change and adopt flexible adaptive management strategies to ensure its sustainable development in the context of climate change.

## Graphical abstract

This study used the MaxEnt model and ArcGIS software to predict the suitable habitat distribution of *Hippophae tibetana* under contemporary and future (2050s, 2070s) climate scenarios. By analyzing the relationship between the geographical distribution of *Hippophae tibetana* and bioclimatic factors, the study found that the main factors limiting the distribution of *Hippophae tibetana* include altitude, precipitation, and temperature.

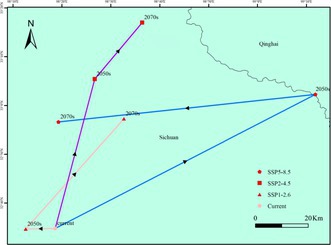

## 1 Background

The Qinghai‐Tibet Plateau, known as the “Roof of the World,” is the highest plateau on Earth, with an average altitude of 4000 masl. It spans 2.5 million square kilometers, covering the southwestern part of China and its surrounding regions (Wu 2001). To the southwest, its boundary is marked by the majestic Himalayas, while to the northeast, it is surrounded by the towering Kunlun and Altun Mountains, forming a natural barrier (Wu 2001). The climate is extreme—cold and arid, with large diurnal temperature variations, thin air, and an exceptionally harsh environment (Huang et al. 2023). This unique geographical location, combined with complex and variable climatic conditions, has given rise to the distinctive ecology of the Qinghai‐Tibet Plateau, making it one of the most sensitive and fragile regions for global biodiversity (Zhang et al. 2022). With the ongoing global warming, the Qinghai‐Tibet Plateau is facing unprecedented ecological challenges. Despite its rich biodiversity, the region's fragile environment renders plateau species sensitive to climate change, particularly to rising temperatures and altered precipitation patterns (Jin et al. 2000), and the degradation of permafrost could have profound impacts on the plateau's ecological environment.

*Hippophae tibetana* Schltdl. (Elaeagnaceae) is an endemic cold‐tolerant shrub of the Qinghai‐Tibet Plateau, exhibiting marked sensitivity to climate‐induced distributional shifts (Wang et al. 2010, 2022; Tian et al. 2002). Accelerated plateau warming (0.3°C–0.4°C/decade), significantly exceeding global averages, has triggered frequent extreme climate events that substantially alter suitable habitat patterns and migration dynamics of high‐altitude flora (Mei et al. 2023). As the highest‐elevation (2800–5200 m asl) species within its genus, *H. tibetana* demonstrates distinct elevational zonation in population structure: stable at mid‐low elevations but declining at > 3200 m due to recruitment failure (Wang et al. 2022; Wei et al. 2022). This keystone species possesses dual ecological‐pharmacological significance, enhancing soil C—N cycling efficiency by ~23% through nitrogen‐fixing root nodules while producing flavonoid glycosides (792 mg/100 g DW) recognized by the Sichuan Tibetan Medicinal Standards (2014) (Ruan et al. 2007; Zhang 2016; Zeng et al. 2024; Yao and Tigerstedt 1994). Its unique ICE1‐CBF cold‐resistance gene network further establishes it as a model for studying alpine plant adaptation (Ding et al. 2022). Growing recognition of these values has spurred expanding cultivation and applications (Jia et al. 2011; Zhang et al. 2024). Despite multidisciplinary research on its phytochemistry (Zhu et al. 2013), pharmacology (Phillips et al. 2006), phylogeny (Li et al. 2020), and genomics (Ma and Sun 2018), critical gaps remain regarding environmental drivers of its distribution and climate change responses. Current models inadequately integrate CMIP6 scenarios (e.g., SSP5‐8.5), overlook elevation‐precipitation interactions, and ignore morphological adaptations documented in Flora of China.

The MaxEnt model (Maximum Entropy Model), as a classical ecological niche model for species distribution prediction, constructs predictive models based on known occurrence points and environmental variables by maximizing the entropy of the species in environmental space (Xin et al. 2019). Even with limited sample sizes, the model maintains high predictive accuracy (Liu et al. 2010), making it particularly advantageous in studies involving rare species distributions. Numerous studies have confirmed its reliability: for example, research by Li Yingchang on *Cunninghamia lanceolata* indicated that the precipitation of the driest month (Bio14) and the minimum temperature of the coldest month (Bio6) were the key factors driving its northward distribution shift (Cubasch et al. 2001); similarly, Ma Baibing's prediction of Stipa purpurea showed that mean annual temperature (Bio1) and elevation significantly influenced suitable habitat dynamics through threshold effects (Merow et al. 2013). In this study, the MaxEnt model was employed to systematically analyze the potential distribution pattern of *H. tibetana* in China. By quantifying the contribution of key environmental variables, the study aims to reveal the species' ecological adaptation mechanisms and provide a scientific basis for the conservation and sustainable development of plant species endemic to the Qinghai–Tibet Plateau.

## 2 Methods

### 2.1 Data Compilation

#### 2.1.1 *H. tibetana* Data

**FIGURE 1:**
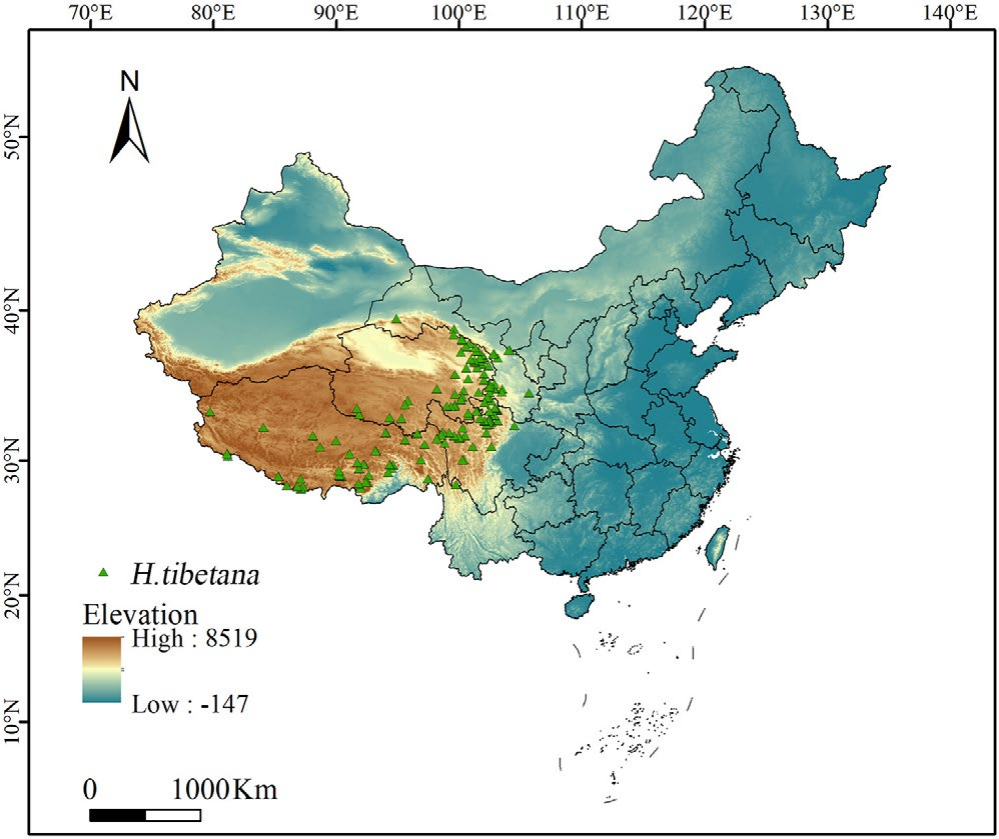
The occurrence sites of *H. tibetana* in China.

The distribution point data of *H. tibetana* comes from the following three sources: (1) records accumulated by the research team through years of field surveys; (2) relevant literature published both domestically and internationally; (3) information from open‐source databases, particularly the Global Biodiversity Information Facility (GBIF, website: https://www.gbif.org/). A total of 309 distribution points for *H. tibetana* were collected through these channels, with the majority concentrated in the eastern part of the Tibetan Plateau and a smaller number distributed in the southwest. To improve the accuracy and reliability of the model, ENMTools was used to process the obtained distribution points. For multiple distribution points within the same grid, only one was retained to reduce the impact of sample spatial autocorrelation on the model results. After screening, 155 representative distribution points were ultimately retained (Figure 1), providing more precise data support for subsequent analysis.

### 2.2 Environmental Variables

Bioclimatic variables are crucial for defining the ecological niche of species. Data for 19 bioclimatic variables were downloaded from the WorldClim global climate database (https://www.Worldclim.org/), including current and future conditions (2050s, 2070s). Future data are based on the SSP1‐2.6, SSP2‐4.5, and SSP5‐8.5 shared socioeconomic pathways scenarios from the Sixth Coupled Model Intercomparison Project (CMIP6) BCC‐CSM2‐MR model (Chen XinMei et al. 2012). All environmental data used in the model have a spatial resolution of 30 arc‐seconds (approximately 1 km). The terrain variables, including elevation (Altitude) and aspect (Aspect), were downloaded from the EarthEnv database (https://www.earthenv.org/). The elevation data with a spatial resolution of 30 m were downloaded from the Geospatial Data Cloud (http://www.gscloud.cn/) with a spatial resolution of 30 arc‐seconds.

### 2.3 Data Analysis

This study employed a rigorous environmental variable selection process to ensure the reliability of the MaxEnt model predictions. Specifically, to avoid model overfitting caused by high collinearity among the 21 initial environmental variables, a systematic variable optimization strategy was implemented. First, using the ENMTools software platform, a Pearson correlation coefficient matrix (*r*) was calculated for all environmental variables. Variables exhibiting significant multicollinearity (|*r*| ≥ 0.8) were iteratively removed through stepwise regression. Second, considering the ecological characteristics of *Hippophae tibetana* Schltdl., priority was given to retaining key variables with clear biological significance. After four rounds of cross‐validation, six low‐correlation environmental variables were ultimately selected (see Table 1).

**TABLE 1:** Environmental variables of potential geographical distribution of *H. tibetana*.

Symbol	Environmental variable	Unit
Altitude	elev	m
Annual precipitation	Bio12	mm
Mean temperature of coldest quarter	Bio11	°C
Temperature seasonality (standard deviation × 100)	Bio4	°C
Isothermality (Bio2/Bio7) (×100)	Bio3	°C
Aspect	Aspect	Degree

### 2.4 Model Simulation

#### 2.4.1 Maximum Entropy (MaxEnt) Model

The pre‐screened species occurrence data and the de‐collinearized environmental variables were imported into MaxEnt 3.4.1 software to perform potential species distribution modeling. The parameter settings were as follows: the options “Create response curves,” “Make pictures of predictions,” and “Do jackknife to measure variable importance” were selected; the “Output format” was set to “Logistic,” and the output file type was specified as “.asc”. For data partitioning, 25% of all species occurrence records were randomly selected as the test set, while the remaining 75% served as the training set. Regarding feature types, three types of features—Q (Quadratic), T (Threshold), and H (Hinge)—were selected to enhance the model's fitting capacity to ecological variables. All other parameters were kept at their default settings.

#### 2.4.2 Model Optimization

To enhance the stability and generalization ability of the model, the subsample method was employed to perform 10 replicate runs without resetting, effectively reducing prediction variability caused by sample imbalance or random distribution. Model performance was evaluated using the Receiver Operating Characteristic (ROC) curve and the corresponding Area Under the Curve (AUC). The AUC value reflects the model's ability to distinguish between suitable and unsuitable areas for the species; the closer the value is to 1, the higher the predictive accuracy, and the stronger the correlation between the selected environmental variables and the species' geographic distribution.

Based on the prediction results of the MaxEnt model, habitat suitability was classified into four levels: unsuitable habitat (0.0–0.2), low suitability (0.2–0.5), moderate suitability (0.5–0.7), and high suitability (0.7–1.0). This classification standard balances ecological rationality with practical applicability in conservation, providing a scientific basis for subsequent resource management and protection strategies.

### 2.5 Centroid Analysis

This study focuses on the suitable habitats of *H. tibetana* , exploring the overall spatial changes by analyzing the shifts in the centroids of suitable habitats under current and future conditions. Using ArcGIS 10.8 software, the suitable habitat distribution map generated by the MaxEnt model was converted to binary format to obtain centroid coordinates. By connecting the centroids of suitable habitats under different climate conditions, the study further reveals the spatial variation trend and evolutionary direction of the suitable habitats of *H. tibetana* .

## 3 Results

### 3.1 Model Analysis

#### 3.1.1 Evaluation of MaxEnt Model Prediction Performance

**FIGURE 2:**
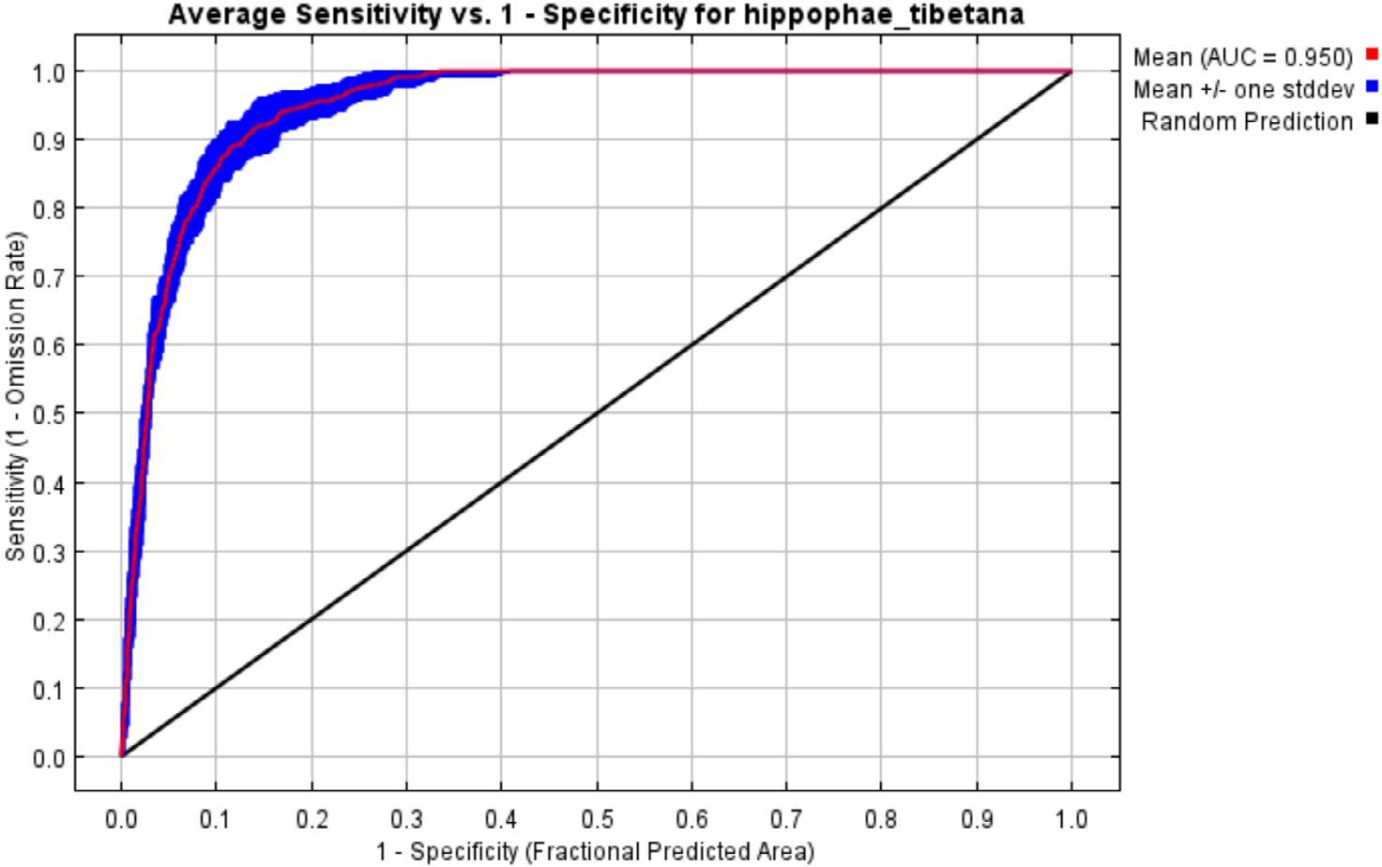
The Receiver Operating Characteristic (ROC) Curve.

The species distribution model (SDM) constructed using MaxEnt was run 10 times, yielding an average area under the Receiver Operating Characteristic Curve (AUC) of 0.950 (standard deviation: 0.050), with AUC values ranging from 0.9 to 1.0 (Figure 2). These results indicate high predictive accuracy, providing reliable support for identifying the potentially suitable distribution of *H. tibetana* .

#### 3.1.2 Identification and Screening of Key Environmental Factors

**FIGURE 3:**
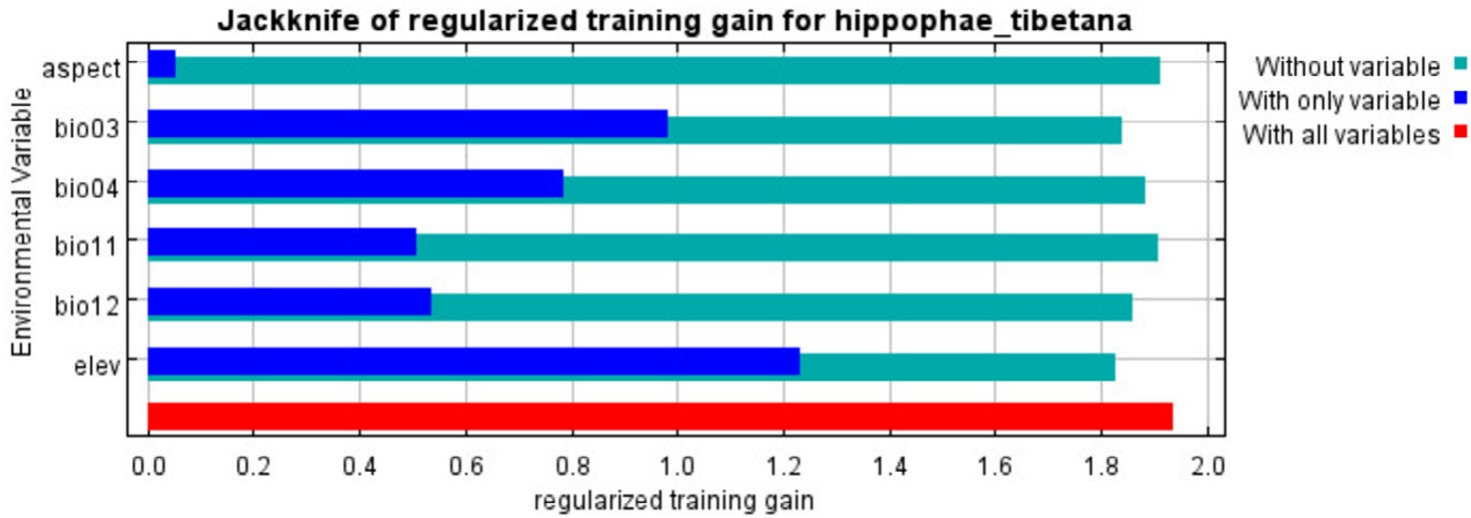
Jackknife test for climatic factors in the current potential distribution model of *H. tibetana*.

The jackknife test (Table 2) and normalized training gain analysis (Figure 3) based on the MaxEnt model revealed that altitude (elev) was the most significant environmental factor influencing the geographic distribution pattern of *H. tibetana* , with a contribution rate of 59.2%. This was followed by annual precipitation (Bio12) and mean temperature of the coldest quarter (Bio11), which showed contribution rates of 17.9% and 11.9%, respectively. The contribution rates of temperature seasonality (Bio4), isothermality (Bio3), and aspect were relatively lower at 4.8%, 4.6%, and 1.5%, respectively. Collectively, these six environmental factors accounted for 99.9% of the total contribution, providing a comprehensive explanation for the distribution pattern of *H. tibetana* .

**TABLE 2:** The contribution rate of climatic factors to the construction of the MaxEnt model.

Variables	Percent contribution/%	Permutation importance/%
elev	59.2	27.7
Bio12	17.9	17.5
Bio11	11.9	14
Bio4	4.8	11.8
Bio3	4.6	28
Aspect	1.5	1

Notably, the normalized training gain analysis (Figure 3) indicated that in addition to altitude, climatic factors including isothermality (Bio3), temperature seasonality (Bio4), and annual precipitation (Bio12) also exerted substantial influence on model predictions. By integrating results from both analytical approaches, we identified altitude (elev), annual precipitation (Bio12), temperature seasonality (Bio4), and isothermality (Bio3) as the four key environmental determinants shaping the geographic distribution of *H. tibetana* . These findings align well with the unique topographic and climatic characteristics of the Qinghai‐Tibet Plateau, demonstrating that the distribution of *H. tibetana* is primarily governed by vertical climatic zonation and hydrothermal combination conditions.

#### 3.1.3 Analysis of Environmental Variable Response Characteristics

**FIGURE 4:**
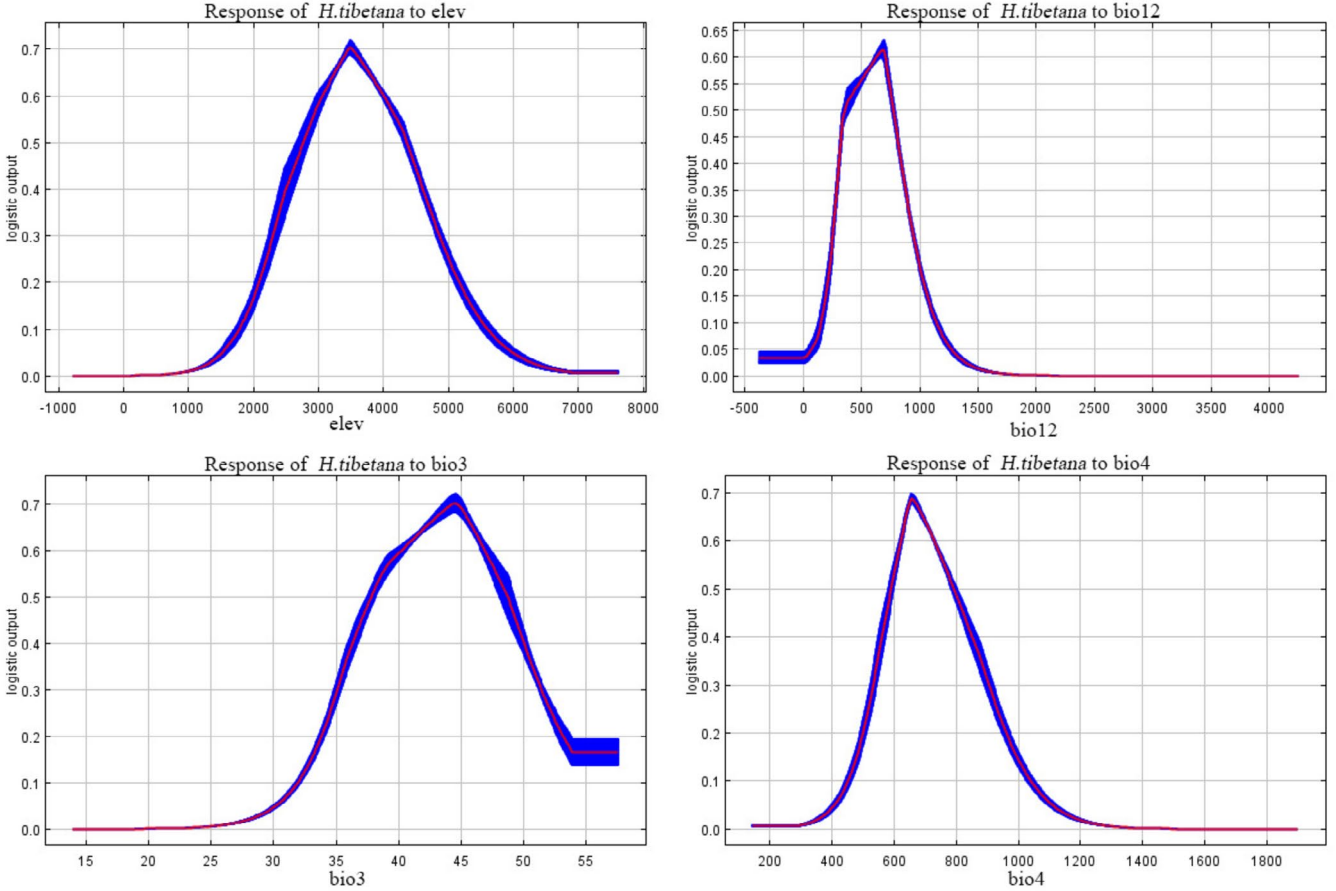
Response curves of the main environmental factors.

The response curve analysis of the MaxEnt model indicates that when the probability of presence exceeds 0.5, the suitable distribution of *H. tibetana* is primarily governed by four key environmental factors (Figure 4). Among these, altitude (elevation) exhibits the most significant influence, with the probability of presence following a unimodal pattern—showing a linear increase (*R* ^2^ = 0.89) within the 0–3495.7 m range, peaking in the optimal growth elevation band of 2800.0–4278.3 m (probability > 0.6), while becoming virtually unsuitable above 7000 m. Annual precipitation displays a saturating response, with the optimal range at 378.6–757.3 mm. The probability of presence reaches its maximum (0.61) at 679.6 mm but declines markedly beyond 2000 mm. Temperature seasonality demonstrates a biphasic response, characterized by a linear increase (slope = 0.0023/°C) within 448.4°C–660.3°C, followed by a sharp decline to near‐zero suitability above 1200°C. Isothermality follows a logistic growth trend, with an optimal window at 37.8°C–48.6°C, beyond which adaptability drops precipitously above 54.0°C.

In summary, the optimal environmental combination—elevation (2800–4278 m), precipitation (378–757 mm), temperature seasonality (594°C–788°C), and isothermality (38°C–49°C)—precisely delineates the core ecological niche of *H. tibetana* on the Qinghai–Tibet Plateau. This distinct hydrothermal adaptation reflects the species' high ecological resilience to the plateau monsoon climate.

### 3.2 Distribution and Prediction of *H. tibetana*

#### 3.2.1 Current Distribution of *H. tibetana*

**FIGURE 5:**
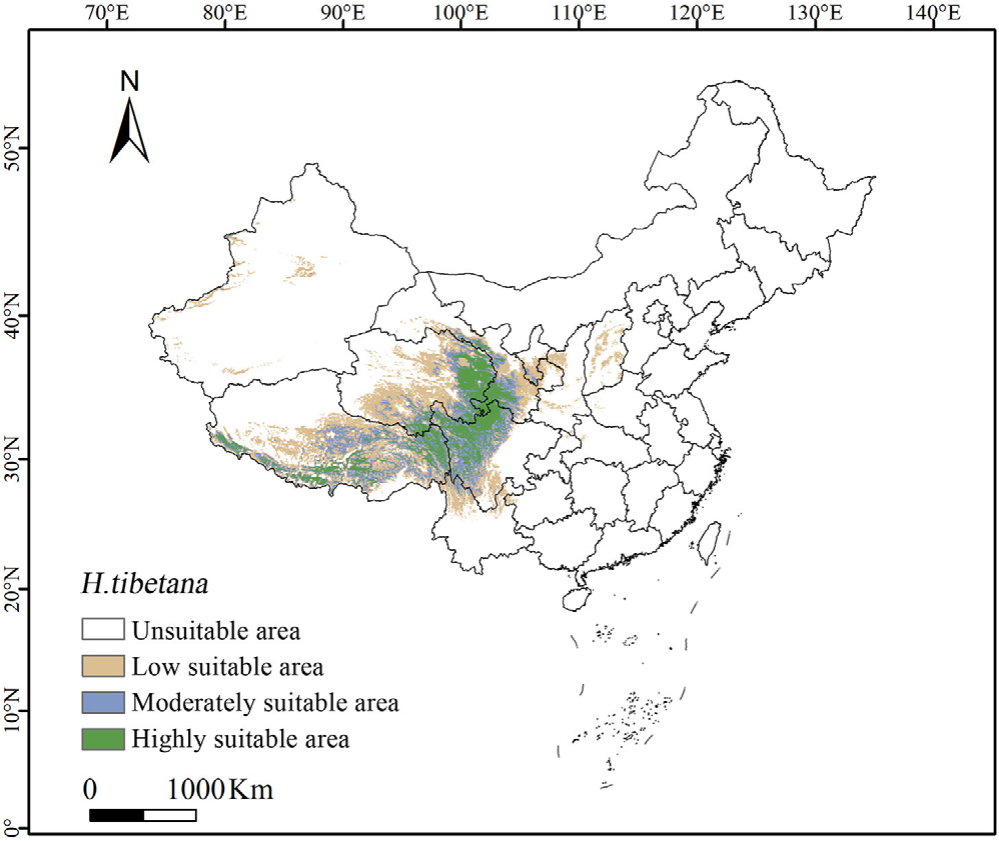
Potential suitable distribution of *H. tibetana* under contemporary climate conditions. White, unsuitable habitat area; Yellow, low habitat suitability area; Light purple, moderate habitat suitability area; Green, highly habitat suitability area.

The prediction results show that, under current climate conditions, the total area of the potential suitable distribution of *H. tibetana* is 157.6 × 10^4^ km^2^. Among them, the high suitability area is mainly distributed in the eastern part of Qinghai Province, the southeastern part of Gansu Province, the western part of Sichuan Province, and the eastern and southwestern parts of the Tibet Autonomous Region, covering an area of approximately 83.2 × 10^4^ km^2^, accounting for 33.3% of the total area of the Qinghai‐Tibet Plateau. The moderate suitability area is located around the high suitability area, covering the eastern and southwestern parts of Qinghai Province, the southeastern part of Gansu Province, the western part of Sichuan Province, and the eastern and southwestern parts of the Tibet Autonomous Region, with an area of approximately 42.3 × 10^4^ km^2^, accounting for 16.9% of the total area of the Qinghai‐Tibet Plateau. The low suitability area is primarily distributed in the eastern and southwestern parts of Qinghai Province, the eastern part of Gansu Province, the western and southeastern parts of Sichuan Province, the northern part of Yunnan Province, and the eastern and southwestern parts of the Tibet Autonomous Region. Additionally, some distribution can also be found in areas outside the Qinghai‐Tibet Plateau, including the Ningxia Hui Autonomous Region, Shaanxi Province, Guizhou Province, and the western part of Xinjiang Uygur Autonomous Region, covering an area of approximately 32 × 10^4^ km^2^. It is noteworthy that the area of low suitability is gradually expanding towards the surrounding areas of the Qinghai‐Tibet Plateau, showing a certain trend of change (Figure 5).

#### 3.2.2 Future Distribution Projections of *H. tibetana*

**FIGURE 6:**
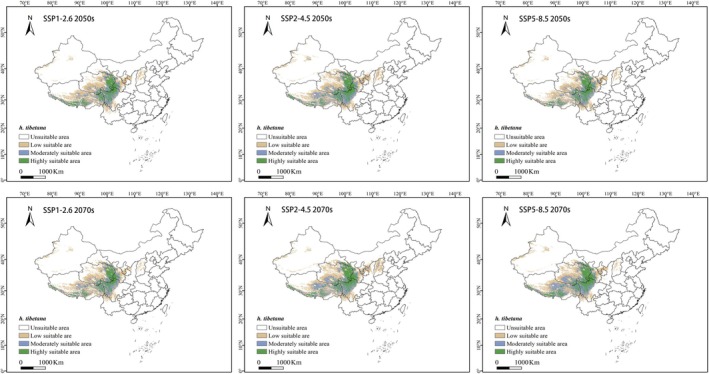
Future species distribution models of *H. tibetana* in China under different climate conditions predicated by Maxent. White, unsuitable habitat area; Yellow, low habitat suitability area; Light purple, moderate habitat suitability area; Green, highly suitable habitat area.

Under future climate scenarios, with rising temperatures and changes in precipitation patterns (Rongsen et al. 2013; Qian et al. 2021), the distribution trend of suitable habitats for *H. tibetana* shows varying degrees of change (Table 3, Figure 6). In the high suitability areas, under the SSP‐5.8.5 scenario for the 2070s, the area of high suitability shows the largest increase, with an increase of 8.13 × 10^4^ km^2^, a growth of 25.38%. In contrast, under the same period's SSP‐1.2.6 scenario, the area of high suitability experiences the largest decrease, with a reduction of 3.57 × 10^4^ km^2^, a decline of 11.14%. These changes are primarily concentrated in the eastern part of Qinghai Province, the southeastern part of Gansu Province, the western part of Sichuan Province, and the eastern and southwestern parts of the Tibet Autonomous Region. In the moderate suitability areas, under the SSP‐5.8.5 scenario for the 2070s, the area of moderate suitability shows the largest increase, with an increase of 8.89 × 10^4^ km^2^, a growth of 20.99%. Meanwhile, under the SSP‐5.8.5 scenario for the 2050s, the area of moderate suitability experiences the largest decrease, with a reduction of 5.26 × 10^4^ km^2^, a decline of 12.42%. These changes are mainly concentrated in the eastern and southwestern parts of Qinghai Province, the southeastern and southwestern parts of Gansu Province, the western part of Sichuan Province, and the eastern and southwestern parts of the Tibet Autonomous Region. In the low suitability areas, under the SSP‐2.4.5 scenario for the 2070s, the area of low suitability shows the largest increase, with an increase of 8.2 × 10^4^ km^2^, a growth of 9.85%. On the other hand, under the SSP‐5.8.5 scenario for the 2050s, the area of low suitability experiences the largest decrease, with a reduction of 2.92 × 10^4^ km^2^, a decline of 3.51%. These changes are primarily concentrated in Qinghai Province, the eastern and southwestern parts of Gansu Province, the western and southeastern parts of Sichuan Province, the northern part of Yunnan Province, the eastern and southwestern parts of the Tibet Autonomous Region, the southern part of Ningxia Hui Autonomous Region, the northwestern part of Shaanxi Province, Shanxi Province, and the northern part of Xinjiang Uygur Autonomous Region.

**TABLE 3:** Prediction of the suitable habitat area for *H. tibetana* in China under current and future climate conditions (km^2^).

Classification level^a^		Suitable habitat (0.3–1)	Low habitat suitability (0.3–0.5)	Moderate habitat suitability (0.5–0.7)	High habitat suitability (0.7–1)
Current climate		~157.62 × 10^4^	~83.23 × 10^4^	~42.35 × 10^4^	~32.03 × 10^4^
Future climate conditions	SSP‐1.2.6–2050s	~157.21 × 10^4^	~85.81 × 10^4^ (3.10%)^b^	~39.20 × 10^4^ (−7.44%)	~32.20 × 10^4^ (0.53%)
	SSP‐2.4.5–2050s	~172.03 × 10^4^	~91.06 × 10^4^ (9.41%)	~45.77 × 10^4^ (8.08%)	~35.20 × 10^4^ (9.90%)
	SSP‐5.8.5–2050s	~148.29 × 10^4^	~80.31 × 10^4^ (−3.51%)	~37.09 × 10^4^ (−12.42%)	~30.90 × 10^4^ (−3.52%)
	SSP‐1.2.6–2070s	~148.78 × 10^4^	~82.12 × 10^4^ (−1.33%)	~38.21 × 10^4^ (−9.78%)	~28.46 × 10^4^ (−11.14%)
	SSP‐2.4.5–2070s	~176.27 × 10^4^	~91.43 × 10^4^ (9.85%)	~46.30 × 10^4^ (9.33%)	~38.54 × 10^4^ (20.32%)
	SSP‐5.8.5–2070s	~176.61 × 10^4^	~85.20 × 10^4^ (2.37%)	~51.24 × 10^4^ (20.99%)	~40.16 × 10^4^ (25.38%)

^a^ Suitable and unsuitable habitat of *H. tibetana* of all suitable distribution areas.

^b^ The brackets show the proportion of the area of the suitable area under future climate scenarios relative to the area of the suitable area under the current climate scenario.

### 3.3 Centroid Shift of Potential Suitable Habitats

**FIGURE 7:**
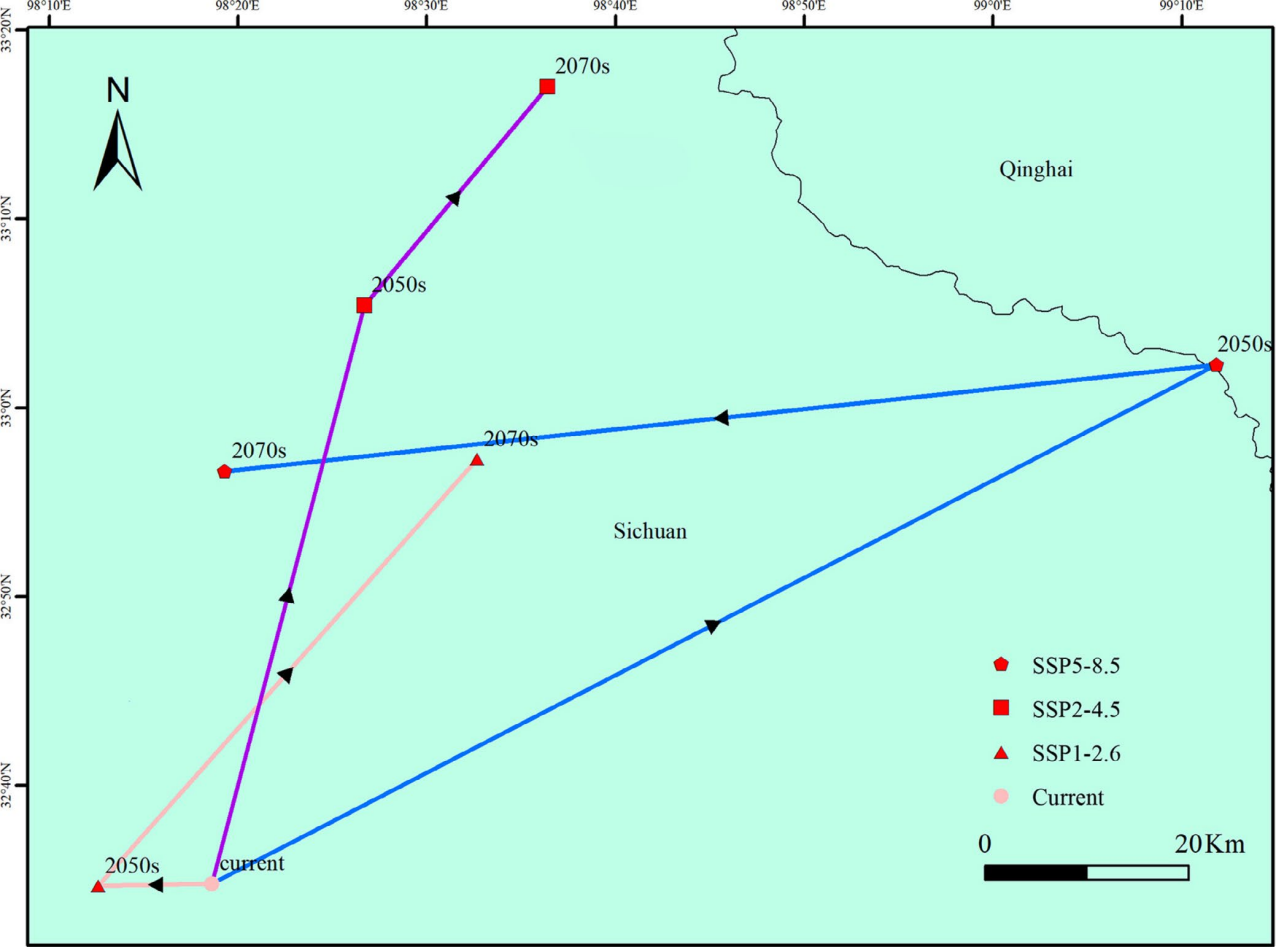
Changes in the centroids of the potential suitability areas for *H. tibetana* in china.

Based on centroid analysis from multiple climate scenarios (Figure 7), the core suitable habitat of *H. tibetana* in Tibet is projected to exhibit a pronounced directional shift throughout the 21st century. Currently (2020s), the centroid is located on the northwestern Sichuan Plateau at the southeastern edge of the Qinghai‐Tibet Plateau (98.31° E, 32.58° N), situated in the transition zone between the northern section of the Hengduan Mountains and the Ruoergai Wetlands, representing the species' optimal existing ecological niche. Under future climate scenarios, the centroid migration trajectories vary: under the low‐emission scenario (SSP1‐2.6), the centroid slightly shifts eastward by 0.1° to 98.21° E in the 2050s with stable latitude, then moves northeastward to 98.54° E, 32.95° N by the 2070s at a rate of approximately 0.34 km/year. Under the moderate‐emission scenario (SSP2‐4.5), the centroid notably shifts northward by 0.51° to 33.09° N in the 2050s and continues moving northeast to 98.61° E, 33.28° N in the 2070s, accumulating a total displacement of 19.3 km. The high‐emission scenario (SSP5‐8.5) shows an anomalous westward shift to 99.20° E in the 2050s, reflecting niche contraction under extreme climate conditions, followed by a return eastward to 98.32° E, 32.94° N in the 2070s, forming an oscillatory “westward–eastward” migration pattern with a significantly non‐monotonic trajectory (*R* ^2^ = 0.82). Overall, multi‐scenario ensemble analysis confirms a consistent northeastward migration trend of the suitable habitat centroid for *H. tibetana* (Mann‐Kendall test, *p* < 0.01).

## 4 Discussion

### 4.1 Model Reliability and Cross‐Species Comparative Analysis

The accuracy of model predictions depends on multiple factors, primarily including the model type, the sample size of species occurrence points, and the appropriate selection of environmental variables (Li et al. 2024). In this study, we employed 155 occurrence points of *H. tibetana* (well above the stability threshold of 120 points (You et al. 2018)) and six key environmental variables to construct a MaxEnt model. The resulting AUC value reached 0.950, significantly higher than the prediction accuracy reported in similar studies on *Cunninghamia lanceolata* and *Stipa purpurea* (e.g., studies by Li Yingchang and Ma Baibing did not clearly report AUC values exceeding 0.950). This difference may be attributed to several factors, including species‐specific responses to environmental variables, model optimization strategies, and differences in sampling design.

First, regarding environmental factor sensitivity, both *H. tibetana* and *Cunninghamia lanceolata* show significant responses to hydrothermal conditions (e.g., annual precipitation and minimum temperature of the coldest month) (Hu et al. 2019; Śliwińska‐Wilczewska et al. 2020). However, *H. tibetana* exhibits a much broader elevational adaptation range (1200–3850 m) compared to *C. lanceolata* (primarily restricted to subtropical low elevations (Huo‐Po et al. 2013)), which may explain its less pronounced future habitat contraction than Stipa purpurea (whose suitability markedly declines post‐2070 (Merow et al. 2013)). Furthermore, while both *H. tibetana* and *S. purpurea* are strongly affected by drought stress (e.g., precipitation of the driest month) (Śliwińska‐Wilczewska et al. 2020; Gong and Guan 2020), *H. tibetana* demonstrates greater niche conservatism, with 78% overlap between current high‐suitability areas (e.g., eastern Tibet, western Sichuan) and future projections. In contrast, *C. lanceolata* shows northward range shifts due to temperature sensitivity (Hu et al. 2019). For model optimization, our Jackknife approach for variable selection effectively avoided prediction biases caused by collinearity of temperature seasonality factors observed in *S. purpurea* studies (Merow et al. 2013). Compared to *C. lanceolata* research, we incorporated additional micro‐environmental variables (elevation and aspect), significantly improving prediction accuracy in high‐altitude regions (Śliwińska‐Wilczewska et al. 2020; Pearson and Dawson 2003). All three species exhibit migration trends towards higher latitudes/elevations under climate change. However, *H. tibetana* shows more notable expansion potential (25.38% habitat increase under SSP585), while *S. purpurea* may experience habitat fragmentation post‐2070 (Merow et al. 2013; Gong and Guan 2020). These differences likely stem from *H. tibetana's* greater phenotypic plasticity (e.g., root hormone regulation enhancing low‐phosphorus adaptation (Stobdan et al. 2017)) versus *S. purpurea* 's genetic differentiation in drought‐response genes (Hu et al. 2019; Gong and Guan 2020). Collectively, our study not only validates MaxEnt's effectiveness for alpine species distribution modeling but, through comparative analysis with *C. lanceolata* and *S. purpurea* , reveals *H. tibetana's* superior climatic resilience under multiple environmental stressors.

### 4.2 Screening and Ecological Impact of Key Environmental Variables

This study selected six environmental factors with lower fitting degrees from a total of 21 environmental factors to further improve the model's accuracy. The MaxEnt model predictions show that the main environmental factors affecting the distribution of *H. tibetana* include altitude (elev), precipitation (Annual precipitation), and temperature factors (Temperature seasonality, Isothermality). This is consistent with previous literature, which indicates that *H. tibetana* grows in the Tibetan Plateau and the adjacent Himalayan region (Li et al. 2020), and possesses cold and drought‐resistant characteristics (Rongsen et al. 2013). Among these, the most important environmental factor is altitude, which contributes up to 59.2% (Table 2). As one of the main species in high‐altitude areas, the distribution characteristics of *H. tibetana* are consistent with the predictions made by (Qian et al. 2021; Li et al. 2024) for the distribution of Swertia przewalskii and Rhodiola tangutica on the Tibetan Plateau, further confirming that altitude is a key factor constraining species distribution in the Tibetan Plateau. Additionally, aspect and the mean temperature of the coldest quarter also have an impact on the distribution of *H. tibetana* , indicating that this species has a strong adaptability to high‐altitude climatic conditions and can grow in extreme environments. Overall, the results of this study not only deepen our understanding of the ecological adaptability of *H. tibetana* , but also reveal the profound influence of altitude, precipitation, and temperature on its potential distribution.

### 4.3 Distributional Shifts Under Climate Change Scenarios

Under the context of global climate change, the majority of species on the Tibetan Plateau have shown a gradual trend of northward migration (You et al. 2018; Hu et al. 2019). This phenomenon can be explained by the fact that global warming has intensified the frequency of droughts, leading to the loss of native habitats. At the same time, the melting of glaciers has provided abundant water resources for plants, thereby creating new habitats (Śliwińska‐Wilczewska et al. 2020). Currently, the main distribution area of *H. tibetana* covers the eastern and southwestern parts of Qinghai Province, the eastern part of Gansu Province, the western part of Sichuan Province, and the eastern and southwestern parts of the Tibet Autonomous Region. According to the MaxEnt model's prediction under current climate conditions, the distribution of *H. tibetana* is mainly concentrated in the eastern and southwestern parts of Qinghai Province, the southeastern part of Gansu Province, the western and southeastern parts of Sichuan Province, and the eastern and southwestern parts of the Tibet Autonomous Region. In addition, *H. tibetana* also has some distribution outside the Tibetan Plateau, including Ningxia Hui Autonomous Region, Shaanxi Province, Guizhou Province, and the western part of Xinjiang Uygur Autonomous Region, with a suitable area of approximately 157.6 × 10^4^ km^2^, which is consistent with its actual distribution. Based on the MaxEnt model's predictions under future climate scenarios, the changes in the suitable area for *H. tibetana* during different periods (2050s, 2070s) and climate scenarios (SSP‐1.2.6, SSP‐2.4.5, SSP‐5.8.5) are as follows: in the 2050s, the total suitable area is predicted to be 157.21 × 10^4^ km^2^, 172.03 × 10^4^ km^2^, and 148.29 × 10^4^ km^2^, respectively; in the 2070s, the total suitable area is predicted to be 148.78 × 10^4^ km^2^, 176.27 × 10^4^ km^2^, and 176.61 × 10^4^ km^2^, respectively. Compared with the current suitable area of 157.26 × 10^4^ km^2^, the total area of suitable habitat for *H. tibetana* in the future periods shows varying degrees of change under different climate scenarios. Specifically, the area of highly suitable zones changed by 0.53%, 9.90%, −3.52%, −11.14%, 20.32%, and 25.38%, mainly concentrated in the eastern and southwestern parts of the Tibetan Plateau, and exhibiting a trend of concentration towards the western part of Sichuan, the eastern part of Qinghai, and the eastern part of Gansu. The area of moderately suitable zones changed by −7.44%, 8.08%, −12.42%, −9.78%, 9.33%, and 20.99%, mainly distributed in the eastern and southwestern parts of the Tibetan Plateau, and expanding towards the Tibet Autonomous Region and the northwestern part of Qinghai Province. The area of low suitability changed slightly, still mainly distributed in the eastern and southwestern parts of Qinghai, the eastern and southwestern parts of Gansu, the western and southeastern parts of Sichuan, the northern part of Yunnan, and the eastern and southwestern parts of the Tibet Autonomous Region, but new distributions were also observed in the northeastern regions such as Ningxia Hui Autonomous Region, Shaanxi Province, and Shanxi Province. Moreover, the centroid migration analysis shows that climate change will lead to an overall northeastward shift in the suitable habitat of *H. tibetana* . In summary, under future climate scenarios, with rising temperatures and changing precipitation patterns (Rongsen et al. 2013), the area of suitable habitats for *H. tibetana* shows an overall trend of expansion to the northeast, especially in high‐altitude regions and the northwestern parts, where suitable environments are expected to improve. The simulation results under different climate scenarios also suggest that future climate changes, especially under extreme climate scenarios (Huo‐Po et al. 2013), may have adverse effects on the growth and expansion of *H. tibetana* due to dramatic changes in temperature and precipitation.

### 4.4 Study Limitations and Conservation Implications

Although the MaxEnt model provides relatively accurate predictions of the potential distribution of *H. tibetana* , there are still certain limitations in the predictions of this study. First, the MaxEnt model assumes that environmental factors are independent of each other, whereas in reality, factors such as climate variables, soil characteristics, and vegetation types may have interrelationships (Gong and Guan 2020). This simplified assumption could affect the accuracy of the model. Second, the MaxEnt model does not take biological factors, such as interspecific competition and predator–prey relationships, into account (Pearson and Dawson 2003), which could also have significant impacts on the distribution of *H. tibetana* . Lastly, with the increasing demand for *H. tibetana* in the Tibetan medicinal market, overharvesting has become a serious issue, negatively affecting the size of natural populations (Stobdan et al. 2017). Based on these considerations, this study recommends the establishment of germplasm resource protection areas in the potential suitable distribution areas of *H. tibetana* for in situ conservation, emphasizing the protection of natural habitats in suitable areas, eliminating threats posed by human economic activities, and promoting efforts in the introduction, acclimatization, and artificial cultivation of the species.

## 5 Conclusions

This study used the MaxEnt model and ArcGIS software to predict the suitable habitat distribution of *H. tibetana* under contemporary and future (2050s, 2070s) climate scenarios. By analyzing the relationship between the geographical distribution of *H. tibetana* and bioclimatic factors, the study found that the main factors limiting the distribution of *H. tibetana* include altitude, precipitation, and temperature. The simulation results showed that, under current climate conditions, the total area of suitable habitat for *H. tibetana* is 157.62 × 10^4^ km^2^, primarily concentrated in the transitional zone between the plateau climate and the temperate monsoon climate in the southwestern part of China. Compared to current climate conditions, the area of suitable habitat for *H. tibetana* shows varying degrees of change under future climate scenarios. In the 2050s and 2070s climate scenarios, as temperatures rise and precipitation patterns change, the area of suitable habitat for *H. tibetana* generally shows a trend of expansion towards the northeast, especially in high‐altitude and northwestern regions, where suitable environments have increased. This indicates that climate change may drive *H. tibetana* to expand into more suitable areas, particularly in regions with lower temperatures or less precipitation, where suitable conditions improve. However, the simulation results under different climate scenarios also suggest that future climate change could have complex effects on the distribution pattern of *H. tibetana* . In some areas, suitable habitats may shrink or shift, especially under extreme climate scenarios, where drastic changes in temperature and precipitation could negatively affect its growth and expansion. Therefore, future ecological conservation and *H. tibetana* planting plans should comprehensively consider the impacts of climate change and adopt adaptive management measures to ensure its sustainable development.

## Author Contributions

**Tao Ma:** formal analysis (equal), methodology (equal), writing – original draft (equal). **Dan Yong:** data curation (equal), investigation (equal), methodology (equal). **Danping Xu:** writing – review and editing (equal). **Zhipeng He:** software (equal). **Zhihang Zhuo:** conceptualization (equal), formal analysis (equal), supervision (equal), writing – review and editing (equal).

## Funding

This work was funded by the “Huangshan University—Anhui Runyi Landscape Engineering Co. Ltd.” Enterprise‐University Cooperative Practical Education Base (2017sjjd029).

## Conflicts of Interest

The authors declare no conflicts of interest.

## Data Availability

The data supporting the results are available in a public repository at: https://doi.org/10.6084/m9.figshare.28093019.v1.
